# A Grand Challenge in Aging Interventions: From Mice to Humans

**DOI:** 10.3389/fragi.2020.566651

**Published:** 2020-09-28

**Authors:** Morten Scheibye-Knudsen

**Affiliations:** Center for Healthy Aging, Department of Cellular and Molecular Medicine, University of Copenhagen, Copenhagen, Denmark

**Keywords:** aging, biomarkers, epigenetics, human trials, model organism

Almost a century has passed since Clive McCay discovered that reducing the food intake of his rats increased their lifespan by up to 40% (McCay and Crowell, 1934). Now we know that dozens of interventions extend the lifespan of organisms such as rodents, nematodes, yeast, and fruit flies. Aging is not as static as it once seemed. Clearly, we now know that several conserved molecular changes occur in organisms with age and we have developed interventions in animal models to impact almost all of them. Nevertheless, despite our great push for testing life- and healthspan altering molecules and growing knowledge of the underlying causes of aging, we still do not know if most of our interventions will work in humans. Why is that?

## Biomarkers

A major problem facing the field of aging is measuring the effect of an intervention. In short lived organisms such as fruit flies, nematodes, and yeast, effects are easy to measure simply by investigating how an intervention impacts the lifespan. However, with longer lived organisms this becomes challenging and surrogate markers are therefore needed that reflect biological aging. Some physiological markers, such as grip strength and walking speed, appear to decline with age and predict mortality (Studenski et al., 2011; Ling et al., 2012), however, there is considerable variability in these physiological parameters and their ability to singly reflect biological aging may be limited. Ten years ago, the identification of single biomarkers of aging was a grand challenge when considering trials for aging in humans, however, landmark papers from Hannum et al. (2013) and Horvath (2013) have shown that we can quite accurately measure age by looking at the combined alterations in the epigenetic landscape. In hind sight, it is perhaps not surprising that complex biomarkers are needed for measuring an effect in a highly complex biological system such as aging. In particular, with the rise of massive laboratory generated datasets and machine learning, a wealth of biomarkers based on proteomics, metabolomics, transcriptomics, microbiomic, photographic, and hematological data, have been developed that can be used to measure efficacy of trials (Rist et al., 2017; Bobrov et al., 2018; Ferrucci and Tanaka, 2018; Fleischer et al., 2018; Galkin et al., 2018; Mamoshina et al., 2018). Notably, most of these biomarkers, or aging clocks, not only predict the age of an individual but also the risk of death, i.e., if you are predicted to be older than your real chronological age you have a higher overall mortality risk. Since some people appear to age faster than others, the predicted age from these algorithms is thought to more closely resemble the true biological age of a person. And we can then use these biomarkers to test if we can reduce or reverse the biological age of an individual. With all these tools at our disposal, we have truly moved into an era where biomarkers are no longer an issue.

## The Trial Design

Concurrent with the recent development in biomarkers the first trial targeting aging in humans are now being started. The most promising ongoing trial is the TAME trial, however, the need for testing a significant number of individuals have been a limiting factor for trial designs. This has been the case because trials are often designed for mortality end-points or other relatively rare events for otherwise healthy elderly individuals which necessitates large cohorts. However, as is evident from the described aging clocks we all are subject to the gradual decay of aging. And these biomarkers are remarkably accurate across populations. The measurements are typically off by an average of 2–8 years corresponding to 5–10% of an individual’s lifespan. Considering this error rate, a simple power calculation shows that you need only sixteen volunteers in a randomized double blinded study if we expect to reduce biomarker age from 75 to 70 years. An additional challenge is to determine how long we have to treat a population before we see an effect. We do not want a treatment period that is too short where changes may be acute responses to a treatment. On the other hand, if we treat for too long the interventions become increasingly costly. A trial aimed at reversing thymic aging in elderly men was recently conducted (Fahy et al., 2019). Although there are several serious issues with this study (small cohort, no blinding, no untreated controls, variable clinical intervention across volunteers etc.) only 10 men were recruited and treated for a year and a significant reduction in epigenetic markers of aging was observed. In another study, 70 adult overweight African-Americans were treated for 16 weeks with vitamin-D resulting in a small but significant decrease in epigenetic aging (Chen et al., 2019). It appears that even relatively short treatments may be enough to see signs of age-reduction in humans. In summary, we have all the tools available to begin transitioning to testing in humans.

## The Grand Challenge

I propose a grand challenge to the field: translate interventions from mice to trials in humans! We need to use the knowledge that we are gathering through countless hours working on model organisms into interventions for humans. We need this not only for our mission to treat age-associated diseases broadly, but also simply to yield creditability to our field. Here, our new open access journal *Frontiers in Aging* may become a key outlet for this research.

## So, Where Do We Go From Here?

Twenty years ago, the NIA funded Interventions Testing Program (ITP) was conceived to test interventions in mice with the specific goal of translating the findings to clinical trials in humans (Warner et al., 2000). The program, which investigates the lifespan effect of proposed interventions in genetically diverse mice across multiple centers, has been a massive success with numerous groundbreaking findings perhaps most notoriously the discovery that rapamycin extends the lifespan of mice (Harrison et al., 2009). Nevertheless, the hope of real translation was never completely carried forward to humans even though some trials have been examining the effect of compounds such as rapamycin on age-associated diseases, but not aging itself. To tackle our grand challenge, I propose that the field funds a human interventions testing program that will investigate promising compounds in humans. In my opinion, the human interventions testing program should be designed in a similar way as the ITP with multiple cohorts across geographically and ethnically diverse populations. Given that we have highly accurate biomarkers cohorts can be small, perhaps as little as 50 individuals per cohort and a treatment time of 6 months after which the intervention should be evaluated perhaps with a composite biomarker. If an effect is observed, another 6 months will be added to the treatment time and another evaluation will be performed. If an effect is still observed another 6 months treatment will be performed etc. Importantly, following this scheme should allow the evaluation of interventions perhaps even faster than the ITP. Obviously, this is simply a possible trial design and others are undoubtedly at least as valuable. The important notion is that we start translation to humans.

## Should We Forget Other Models?

No! As is the case in the pharmaceutical industry, mice, and other animal models are critical for preclinical development and mechanistic investigations. We will still need our continuous focus on the basic biology of aging and testing of interventions for safety and pharmacological reasons. Evidently, we are still not able to stop aging in mice or any other organism and significant areas within the biology of aging remain uncharted. I therefore encourage you, dear reader, to submit any intervention study regardless of species to our open access journal.

## Final Thoughts

In the past decades, we have come to realize that aging can be malleable and that there is a glimmer of hope that we may perhaps be able to escape the slow decay and the final rest. Science is the only thing that will get us there.

## Author Contributions

MS-K is the sole contributor of this work.

## Conflict of Interest

The authors declare that the research was conducted in the absence of any commercial or financial relationships that could be construed as a potential conflict of interest.
